# Antigenic Cross-Reactivity Between SARS-CoV-2 S1-RBD and Its Receptor ACE2

**DOI:** 10.3389/fimmu.2022.868724

**Published:** 2022-05-04

**Authors:** Yen-Chung Lai, Yu-Wei Cheng, Chiao-Hsuan Chao, Yu-Ying Chang, Chi-De Chen, Wei-Jiun Tsai, Shuying Wang, Yee-Shin Lin, Chih-Peng Chang, Woei-Jer Chuang, Li-Yin Chen, Ying-Ren Wang, Sui-Yuan Chang, Wenya Huang, Jen-Ren Wang, Chin-Kai Tseng, Chun-Kuang Lin, Yung-Chun Chuang, Trai-Ming Yeh

**Affiliations:** ^1^Department of Medical Laboratory Science and Biotechnology, College of Medicine, National Cheng Kung University, Tainan, Taiwan; ^2^ Leadgene Biomedical, Inc., Tainan, Taiwan; ^3^The Institute of Basic Medical Sciences, College of Medicine, National Cheng Kung University, Tainan, Taiwan; ^4^OmicsLab Co., Ltd., New Taipei City, Taiwan; ^5^Department of Microbiology and Immunology, College of Medicine, National Cheng Kung University, Tainan, Taiwan; ^6^Department of Biochemistry and Molecular Biology, College of Medicine, National Cheng Kung University, Tainan, Taiwan; ^7^Department of Clinical Laboratory Sciences and Medical Biotechnology, College of Medicine, National Taiwan University, Taipei, Taiwan; ^8^SIDSCO Biomedical Co., Ltd., Kaohsiung, Taiwan; ^9^Department of Biomedical Sciences, Chung Shan Medical University, Taichung, Taiwan

**Keywords:** COVID-19, autoantibody, angiotensin converting enzyme 2, monoclonal antibody, molecular mimicry

## Abstract

Severe acute respiratory syndrome coronavirus 2 (SARS-CoV-2) is an emerging virus responsible for the ongoing COVID-19 pandemic. SARS-CoV-2 binds to the human cell receptor angiotensin-converting enzyme 2 (ACE2) through its receptor-binding domain in the S1 subunit of the spike protein (S1-RBD). The serum levels of autoantibodies against ACE2 are significantly higher in patients with COVID-19 than in controls and are associated with disease severity. However, the mechanisms through which these anti-ACE2 antibodies are induced during SARS-CoV-2 infection are unclear. In this study, we confirmed the increase in antibodies against ACE2 in patients with COVID-19 and found a positive correlation between the amounts of antibodies against ACE2 and S1-RBD. Moreover, antibody binding to ACE2 was significantly decreased in the sera of some COVID-19 patients after preadsorption of the sera with S1-RBD, which indicated that antibodies against S1-RBD can cross-react with ACE2. To confirm this possibility, two monoclonal antibodies (mAbs 127 and 150) which could bind to both S1-RBD and ACE2 were isolated from S1-RBD-immunized mice. Measurement of the binding affinities by Biacore showed these two mAbs bind to ACE2 much weaker than binding to S1-RBD. Epitope mapping using synthetic overlapping peptides and hydrogen deuterium exchange mass spectrometry (HDX-MS) revealed that the amino acid residues P463, F464, E465, R466, D467 and E471 of S1-RBD are critical for the recognition by mAbs 127 and 150. In addition, Western blotting analysis showed that these mAbs could recognize ACE2 only in native but not denatured form, indicating the ACE2 epitopes recognized by these mAbs were conformation-dependent. The protein–protein interaction between ACE2 and the higher affinity mAb 127 was analyzed by HDX-MS and visualized by negative-stain transmission electron microscopy imaging combined with antigen-antibody docking. Together, our results suggest that ACE2-cross-reactive anti-S1-RBD antibodies can be induced during SARS-CoV-2 infection due to potential antigenic cross-reactivity between S1-RBD and its receptor ACE2.

## Introduction

Severe acute respiratory syndrome coronavirus 2 (SARS-CoV-2) is a new emerging virus that is rapidly spreading in humans and thus causing the ongoing global coronavirus disease 2019 (COVID-19) pandemic (1). SARS-CoV-2 is a β-coronavirus, a subgroup that is taxonomically very close to SARS-CoV but more distantly related to MERS-CoV and common human CoVs (2). The spike (S) protein of SARS-CoV-2 is a ~180 kDa glycoprotein, which can form a trimeric structure that protrudes from the surface of the viral particle, plays a key role in the recognition of the cell surface receptor angiotensin-converting enzyme 2 (ACE2) (3) and cell membrane fusion (4). The total length of the SARS-CoV-2 S protein contains 1273 amino acids (a.a) arranged into two subunits: the S1 subunit (a.a. 14-685) contains a receptor-binding domain (S1-RBD, a.a. 319-541) that is less conserved between SARS-CoVs and other CoVs, having only a range of 20-64% identity match, whereas the S2 subunit (a.a. 686-1273) mediates viral cell membrane fusion, exhibiting higher sequence identity (~90%) (4–7). The receptor-binding motif (RBM, a.a. 437-507) is a portion of the S1-RBD that makes direct contact with ACE2, whereas S2 subunit mediates subsequent membrane fusion with the host cell membrane (8). The binding of S protein to ACE2 triggers the cleavage between S1 and S2 by host furin and TMPRRS2 proteases, which is responsible for the transition of S2 subunit to the “fusion” conformation to initiate fusion to enable viral entry into cells (9).

Since the binding of S protein to ACE2 is the first step in the process of SARS-CoV-2 infection, it is a key determinant of host cell and tissue tropism of SARS-CoV-2. Indeed, SARS-CoV-2 S1-RBD appears to exhibit improved binding efficiency to human ACE2 compared with that of the 2003 strain of SARS-CoV (3, 10). In addition to the mutation of the S1-RBD which can cause significant variation in the S1-RBD/ACE2 binding affinity (11), the distribution of ACE2 and TMPRRS2 are primary limiting cell-entry factors for the susceptibility of different tissues and cell types to SARS-CoV-2 entry and infection. A list of 28 cellular factors, referred to as SARS-CoV-2 and coronavirus-associated receptors and factors (SCARFs) are identified using single-cell transcriptomics across various human tissues, which are involved in either facilitating or restricting viral entry (9). These cellular factors are also important in determining the potential tissue tropism of SARS-CoV-2.

Analysis of SARS-CoV-2 S glycan reveals that it is heavily glycosylated, providing shielding from antibody recognition, with the exception of the S1-RBD. Intriguingly, the S1-RBD is structurally flexible which can change between an open (up) and closed (down) conformation. While S1-RBD at open conformation is required to be able to interact with ACE2, Cryo-EM study of S protein trimers reveals that on average only ~20% of S1-RBD are in the open state (12–14). Interestingly, the glycosylation of S protein is also involved in the transition between the open vs. closed state of the S1-RBD (15). Since S1-RBD at the open state increases the possibility of being recognized by host antibodies, it is likely that S protein evolved this conformational dynamic change to balance infection and immune evasion (8).

Unlike 2003 SARS, COVID-19 commonly causes tissue damage in non-respiratory organs, such as the heart, liver, kidney, and brain (16, 17). However, what leads to the wide range of clinical pathologies observed in COVID-19 patients is not yet understood. It remains unclear whether these pathological damages are caused by direct SARS-CoV-2 infection of the organs affected or indirect effects by immune responses or comorbidities. In addition to viral and cellular entry factors, many host immune responses have been proposed to contribute to the severity and multiple organs involvement of COVID-19, including dysregulated inflammatory response and autoimmunity (18–21). For example, the development of IgG, IgM, and IgA autoantibodies against ACE2 in patients with COVID-19 has been reported (22–25), and their levels in sera are associated with COVID-19 disease severity (24). However, the mechanisms through which antibodies against ACE2 are induced during SARS-CoV-2 infection are unclear.

In this study, we first confirmed the increase in antibodies against ACE2 in patients with COVID-19 and demonstrated a positive correlation between the amounts of antibodies against ACE2 and S1-RBD. In addition, the antibody binding to ACE2 was significantly decreased in the sera of some COVID-19 patients after preadsorption of the sera with S1-RBD. To confirm that antibodies against S1-RBD can indeed cross-react with ACE2, we immunized mice or rabbits using recombinant S1-RBD generated by bacteria, insect cells, or mammalian cells and found that anti-ACE2 antibodies were also increased in various sources of S1-RBD immune sera. Two monoclonal antibodies (mAbs) that could recognize both S1-RBD and ACE2 were identified. Thus, our results suggest the existence of potential antigenic cross-reactivity between S1-RBD and its receptor ACE2.

## Materials and Methods

### Recombinant Proteins, Peptides, and Patient Serum

C-terminal Twin-Strep-tagged SARS-CoV-2 S1-RBD recombinant proteins (from a.a. 319 to 541 of the S1 protein, YP_009724390) were cloned into pMT/BiP/V5-His B plasmid for S2 cell expression. The purity of SARS-CoV-2 S1-RBD from S2 cell was >90% by using SDS-PAGE analysis. The yield was around 4.6 mg/L. SARS-CoV-2 S1-RBD from *E. coli* and CHO (Cat. No. 61931/62433, Leadgene Biomedical Inc.) as well as different SARS-CoV-2 S1-RBD mutants and S1-RBD peptides were customized, purified and synthesized by Leadgene Biomedical, Inc. Tainan, Taiwan. C-terminal Fc-tagged human ACE2 recombinant protein (ACE2-hFc) was purified from CHO cells (Cat. No. 63333, Leadgene Biomedical Inc.). C-terminal His-tagged human ACE2 (10108-H08H) and other coronavirus S1 proteins (40150-V08B1, 40591-V08H, 40069-V08H, and 40600-V08H) were purchased from Sino Biological (Beijing, China). In addition, commercially available COVID-19-positive (Panel D, n=30) and COVID-19-negative patient sera (Panel E, n=60) were purchased from Access Biologicals (Vista, CA, USA). According to the manufacture, individual donor units used in the cohort have been tested and found negative by tests for antibodies to HIV 1/2, HCV and non-reactive for HBsAg. All testing was performed with kits approved by the FDA. The samples were collected under IRB approved protocols. The commercial COVID-19 positive and negative patient sera were dispensed in Biosafety Level-2 plus (BSL-2+) laboratory. All individuals participating in patient sera dispensation were fully trained according to the compliance policies of the National Cheng Kung University Hospital. Appropriate personal protective equipment was always worn when working with patient sera in the BSL-2+ room. Dispensed patient sera were inactivated at 56°C for 30 min before being used in this study (**Supplementary Table**).

### Immunization and mAb Generation

For the preparation of mouse and rabbit S1-RBD-hyperimmune sera, recombinant proteins (25 μg for mice, 250 μg for rabbits) were emulsified with alum or Incomplete Freund’s Adjuvant (IFA) (Sigma–Aldrich, St Louis, MO, USA). The animals were primed and challenged on days 1, 14 and 21 using alum adjuvant or on days 1, 7 and 14 using IFA. Sera were collected 7 days after the final challenge and stored at -20°C until use. For mAb generation, mice at the Leadgene Biomedical, Inc., facility were immunized with S1-RBD expressed in *E. coli* according to the hybridoma technique as previously described (26, 27). In brief, the splenocytes were fused with mouse myeloma FO cells and selected by modified selected-medium containing hypoxanthine–aminopterin–thymidine (Thermo Fisher Scientific, MA, USA), 15% fetal bovine sera (FBS) (HyClone, Logan, UT) and 2.5% HyBoost (Leadgene Biomedical Inc., Taiwan). Cloning hybridoma cells was performed by limiting dilution. Supernatants of the clones were collected and screened for antibodies against SARS-CoV-2 S1-RBD and ACE2. Hybridoma cells were injected into mouse peritoneal cavities to generate ascites, and the mAbs in ascites were harvested by protein G-Sepharose (GE Healthcare), dialyzed against phosphate-buffered saline (PBS), and stored below -20°C.

### Enzyme-Linked Immunosorbent Assays

Each well of an ELISA plate (Corning Costar, Acton, MA, USA) was coated with 100 μL of antigens (1 μg/mL recombinant protein in PBS or 2 μg/mL bovine serum albumin (BSA)-conjugated peptides in carbonate coating buffer, pH 9.6) for 16 h at 4°C, washed three times with PBST (0.1% Tween 20) and blocked with 5% skim milk for 1 h at 37°C. For the detection of antibody binding to ACE2 in human sera, the ELISA plates were coated with His-tagged ACE2 instead of ACE2-hFc to prevent nonspecific binding of the secondary antibody to human Fc. In contrast, to detect antibody binding to ACE2 in immune sera from mice or rabbits, the ELISA plates were coated with ACE2-hFc to prevent nonspecific binding of anti-His antibodies in immune sera. The sera were diluted in PBST and added to the wells of the ELISA plates, and the plates were incubated for 1 h at 37°C. HRP-labeled secondary antibodies against human (C04047, Croyez, Taiwan), mouse (115-035-062, Jackson ImmunoResearch, West Grove, PA, USA), or rabbit IgG (C04010, Croyez, Taiwan) were diluted 10,000-fold in PBST and used for the detection of bound antibodies. In the competition ELISA, the Abs (1 μg/mL and 25 μg/mL for S1-RBD- and ACE2-hFc-coated plates, respectively) were preincubated with different S1-RBD polypeptides at different doses as indicated, for 1 h at 37°C. Subsequently, the Ab-peptide mixtures were incubated in S1-RBD- or ACE2-hFc (2 μg/mL)-coated plates for another 30 min, washed with PBST and incubated with HRP-labeled secondary antibodies against mouse IgG for another 1 h. For color development, 100 μL of TMB PLUS2 (Kementec Solutions A/S, Denmark) was added to the wells, the plates were incubated for 10 min at 37°C, and the reaction was stopped by the addition of 50 μL of 0.2 M sulfuric acid. The absorbance at 450 nm was determined using an ELISA reader (Multiskan, Thermo Fisher Scientific, Waltham, MA, USA).

### Serum Preadsorption Assay

In the serum preadsorption assay, ELISA strip wells (Corning) were coated with BSA (10 μg/mL, 100 μL), S1-RBD (10 μg/mL, 100 μL), and ACE2-His (1 μg/mL, 100 μL). The strip wells were washed three times with PBST and blocked with 1% BSA in PBS. The sera were diluted in PBS at 1:100 dilution and then added to BSA- or S1-RBD-coated wells. After incubation at 37°C for 1 h, the diluted sera were transferred to ACE2-His-coated wells. Unbound antibodies were discarded after further incubation at 37°C for 1 h. Bound IgG against ACE2 was detected using HRP-conjugated goat anti-human IgG antibody (1:5,000, C04047, Croyez).

### Western Blotting

SARS-CoV-2 NTU13-infected cell lysates provided by NTUH (28) were harvested using RIPA buffer III (Bio Basic Inc., Markham, Ontario, Canada). The cell lysates or recombinant proteins were prepared under reducing or nonreducing conditions prior to loading onto 10% or 12% SDS–PAGE gels for separation as indicated. For non-reducing condition, sample buffers containing 2% SDS and 15% glycerol were added to the samples prior to loading into SDS-PAGE; for reducing condition, sample buffers containing 2% SDS, 15% glycerol and 1% 2-ME were added to the samples, following by heat-denaturing at 95°C for 5 min prior to loading into SDS-PAGE. The separated proteins were transferred onto a PVDF membrane (Pall, Ann Arbor, MI, USA). The membrane was blocked with 5% skim milk in TBST (0.05% Tween 20 in Tris-buffered saline, TBS), incubated with primary antibodies, namely, anti-His antibody (10411, Leadgene Biomedical Inc.), anti-SARS-CoV-2 S1 mAb (GTX635656, GeneTex Inc, Irvine, CA, USA), anti-ACE2 polyclonal antibody (anti-ACE2 pAb; ARG41099, Arigo, Taiwan) or mAbs 127 and 150 overnight at 4°C and detected with HRP-conjugated goat anti-rabbit or goat anti-mouse IgG secondary antibodies (1:10,000 dilution; Leadgene Biomedical) for another 1 h. Detection was then performed using an Enhanced Chemiluminescence Western blotting Kit (Advansta, Menlo Park, CA, USA).

### Immunofluorescent Assay

Antibodies or sera were diluted in 1% BSA containing anti-microbial agent, 0.01% sodium azide. Anti-ACE2 pAb was used as a positive control. A stable clone of ACE2-overexpressing HEK293 (HEK293-ACE2) was generated by transfection with a pcDNA3.1 plasmid which was inserted tag free native sequence of full-length human ACE2 gene (NP_068576.1) within Nhe I and Xho I restriction enzyme sites. HEK293 and HEK293-ACE2 cells were fixed in 0.5% paraformaldehyde and stained with diluted antibodies or sera for 3 h. Subsequently, the cells were washed three times with PBS and then combined with fluorescence-labeled secondary antibodies against mouse, rabbit (C04025 and C04030, Croyez), or human IgG (A-11013, Thermo Fisher Scientific). An EVOS FL Auto 2 Imaging System (Thermo Fisher Scientific) was used for detection.

### Biacore Surface Plasmon Resonance

All SPR measurements in this study were performed using a Biacore T200 (Cytiva/GE Healthcare Life Sciences). The recombinant proteins S1-RBD-His and ACE2-hFc were first covalently immobilized on Sensor CM5 chips (Cytiva/GE Healthcare Life Sciences) *via* amine coupling according to the manufacturer’s protocol. S1-RBD-His was diluted in 10 mM sodium acetate, pH 5.0, to a final concentration of 10 μg/mL and injected into the activated flow cell to obtain an immobilization level at 884.6 and 1047.1 RU. For ACE2-hFc immobilization, a diluted concentration of 30 μg/mL in 10 mM sodium acetate, pH 4.5, was used to reach an immobilization level at 1482.1 RU. For analysis of the binding of mAb to S1-RBD-His, serial dilutions of mAb were injected into the immobilized chip, which contained 884.6 RU of S1-RBD-His, and the concentrations in these dilutions ranged from 256 nM to 8 nM. In the analysis of mAb binding to ACE2-hFc, serial dilutions of mAb with concentrations ranging from 1024 nM to 8 nM were used. All analyte injections were performed at a flow rate of 30 μL/min for 120 s and a dissociation time of 360 s (600 s was used in the assay of ACE2-hFc binding to S1-RBD-His). For regeneration, 10 mM glycine-HCl, pH 2.5 (Cytiva/GE Healthcare Life Sciences), was injected at a flow rate of 30 μL/min for 30 s. The assays were performed using the Kinetic/Affinity wizard, and all the procedures were conducted at 25°C. The binding kinetics were determined using Biacore T200 Evaluation Software version 3 (Cytiva/GE Healthcare Life Sciences).

### Hydrogen Deuterium Exchange Mass Spectrometry Analysis and Peptide Identification

The footprints of mAbs 127 and 150 on S1-RBD and mAb 127 on ACE2 in the presence or absence of mAb were measured by HDX-MS analysis. The protein-antibody complex (15 pmol of antigen and 10 pmol of antibody were pre-incubated at room temperature for 1 hour) were diluted in the exchange buffer (99.9% D_2_O in PBS, pH 7.4) at 1:9 ratio to initiate HD exchange at room temperature. At two time points (5 and 10 min), an aliquot (1.5 pmol of target protein) was aspired and mixed with quenching buffer (to a final concentration of 1.5 M guanidine hydrochloride, 150 mM tris (2-carboxyethyl) phosphine, and 0.8% formic acid). The mixture was immediately loaded onto homemade pepsin column for online digestion. The MS/MS spectra of pepsin-digested fragments were searched against the antigen protein database using the SEQUEST search engine. The HD exchange number of two independent HDX-MS experiments (duplicates) was then averaged and presented as differential levels of HD exchange [(exchanged D in antigen – exchanged D in antigen/antibody)/(exchanged D in antigen/antibody)]. Peptide identification was conducted using Proteome Discoverer software (version 2.2, Thermo Fisher Scientific).

### Negative-Stain Transmission Electron Microscopy Analysis for mAb 127 and ACE2-hFc Image Analysis by 2D Class Averaging

Negative-stain transmission electron microscopy analysis for mAb 127 and ACE2-hFc image analysis by 2D class averaging was performed with a mixture of mAb 127 and ACE2-hFc (the total concentration of sample mixture was 10 ng/mL at 4:1 molar ratio) in PBS. The samples were stained with 1% uranyl acetate and then added to charged carbon-coated grids. The images were taken with a JEM1400 electron transmission microscope (Jeol Ltd., Tokyo, Japan) at 12,000× magnification using a 4k × 4k Gatan 895 CCD camera.

### Structural Prediction and Docking of the Antigen-Binding Fragment of mAb 127 and Antigens

We used AlphaFold2 to create a predicted structure of mAb 127 Fab, and the interaction between mAb 127 Fab and S1-RBD (PDB ID: 7VN) or mAb 127 Fab and ACE2 (PDB ID: 1R42) were modeled by Maestro v10.1 (Schrödinger) docking analysis. AlphaFold2 is a neural network deep learning modeling which was used to predict the structure of proteins (29). It leverages neural networks and multiple alignments to predict structure. The sequence of mAb 127 was inputted with pair_msa option to generate a predicted structure which was further used to perform docking analysis by Maestro software. In brief, Protein Preparation Wizard was used to add hydrogens and created zero-order bonds to metals and disulfide bonds of antigens and mAb 127 Fab. Optimization H-bind assignment was used PROPKA (pH 7.0) and restrained minimization was applied OPLS3e of force field. For protein-protein docking, we chose “antibody” mode to perform the analysis and visualized the result using PyMol version 2.4.1.

### Statistical Analysis

All statistical data were analyzed using unpaired Student’s t test to compare two independent groups or using one-way ANOVA to compare more than two groups. The analyses were performed using Prism software (GraphPad Software Inc., CA, USA). All data are presented as the means ± standard deviations (S.Ds.) from at least two independent experiments. *P < 0.05, **P < 0.01, ***P < 0.001, and ns indicates no significance based on 95% two-tailed confidence intervals.

## Results

### Antibodies Against SARS-CoV-2 S1-RBD in Sera From Patients with COVID-19 Cross-React With ACE2

**Figure 1:**
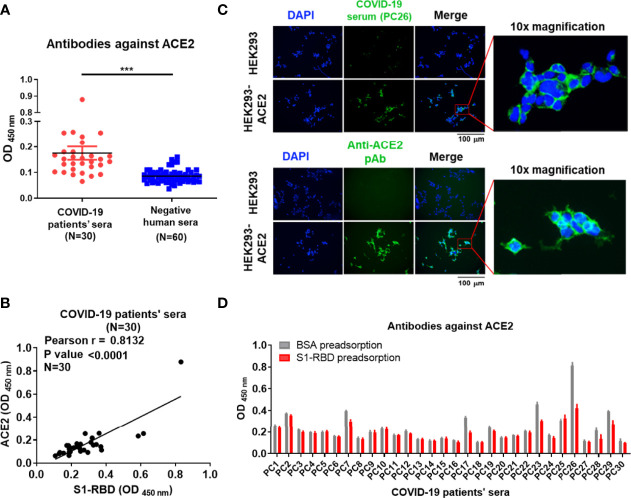
Antibodies against S1-RBD in sera from patients with COVID-19 cross-react with ACE2. **(A)** The binding of sera (1:400 dilution) collected from 30 SARS-CoV-2-infected patients or 60 healthy humans to ACE2 was analyzed by ELISA using ACE2-His-coated plates. **(B)** Correlation of the OD of anti-S1-RBD (x-axis) and anti-ACE2 (y-axis) antibodies in the sera of patients with COVID-19 (N = 30). The binding ability was analyzed by indirect ELISA. **(C)** HEK293 and HEK293-ACE2 cells were fixed and stained with either COVID-19-positive PC26 serum (1:400 dilution) or an anti-ACE2 pAb (5 μg/mL) and then visualized using an immunofluorescent assay. Scale bar = 100 μm. The right images were derived from the original photographs at 10× magnification. The PC26 serum and anti-ACE2 pAb reacted with ACE2 expressing on the surface of HEK293 was visualized by fluorescent secondary antibody in green. All statistical data are presented as the means ± S.Ds. from at least two independent experiments. ***P < 0.001 **(D)** The sera (1:100 dilution) collected from 30 SARS-CoV-2-infected patients were preadsorbed by either BSA or S1-RBD prior to binding to ACE2-coated plates. The binding ability of preadsorbed sera to ACE2 was analyzed by indirect ELISA.

To confirm the presence of antibodies against ACE2 in sera from patients with COVID-19, a panel of 30 commercial serum samples collected from patients with COVID-19 and 60 serum samples from normal individuals were screened for the presence of antibodies against S1-RBD and ACE2 by ELISA. We found that the mean optical density (OD) of antibodies against ACE2 in sera from patients with COVID-19 was indeed significantly higher than that in healthy cohorts (**Figure 1A**). Moreover, a positive correlation was found between the titer of antibodies against the S1-RBD protein and that of antibodies against ACE2 in sera from patients with COVID-19 (Pearson r = 0.8132, p value < 0.0001) (**Figure 1B**). Among the 30 serum samples from patients with COVID-19, PC26 exhibited the highest antibody titer against both ACE2 and S1-RBD. To prevent the analysis from being skewed by this patient, we also analyzed the correlation after omitting the data from PC26. Even though the omission of these data decreased the Pearson r to 0.7049, the p value remained < 0.0001. To further characterize the properties of these ACE2-reactive antibodies in sera from patients with COVID-19, PC26 was used to stain HEK293-ACE2 cells. As shown in **Figure 1C**, antibodies from PC26 were able to bind to HEK293-ACE2 but not wild-type (WT) HEK293 cells, as demonstrated by immunofluorescent assay. A similar staining pattern was found using an anti-ACE2 pAb (**Figure 1C**). To confirm that antibodies against SARS-CoV-2 S1-RBD can indeed cross-react with ACE2, sera from patients with COVID-19 were preadsorbed to S1-RBD or BSA-coated ELISA plates and then tested for anti-ACE2 antibodies by ELISA. Approximately 20% of the sera from patients with COVID-19 (6 out of 30) showed significantly decreased levels of antibody binding to ACE2 after S1-RBD preadsorption **(** **Figure 1D****)**. Notably, the sera of these patients with COVID-19 usually had higher amounts of antibodies against ACE2 before adsorption than the sera of other patients with COVID-19.

### SARS-CoV-2 S1-RBD Immunization Induces Antibody Binding to ACE2

**Figure 2:**
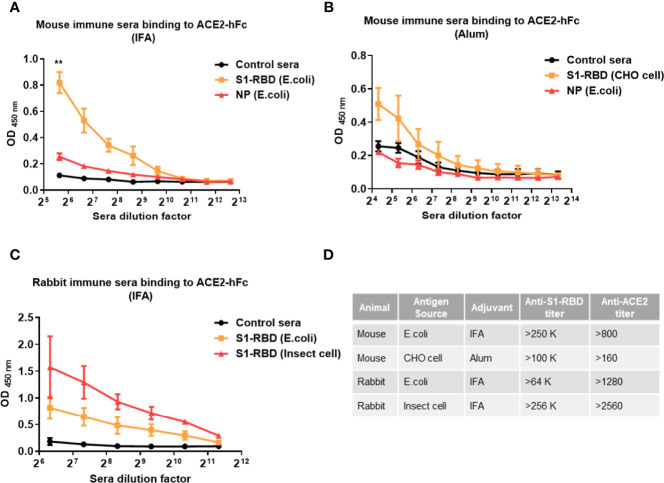
Immunization with SARS-CoV-2 S1-RBD elicits antibodies that cross-react with human ACE2. Mice or rabbits were immunized with recombinant S1-RBD generated from different sources, such as E. coli, mammalian cells (CHO cells) and insect cells (S2), using IFA or alum as indicated. Four mice or rabbits were included in each group for N value **(A–C)** Different dilutions of serum antibodies binding to ACE2 from S1-RBD- or NP-immunized mice or rabbits were measured by ELISA as indicated. In addition, normal mouse or rabbit sera were used as control sera. **(D)** Comparison of antibody titers against S1-RBD or ACE2-hFc in different immune sera samples measured by ELISA. The titer indicates that the highest dilution of end-point titers of sera that still showed a positive reaction. All statistical data are presented as the means ± S.Ds. from at least two independent experiments. **P < 0.01.

To further confirm that SARS-CoV-2 S1-RBD can indeed induce ACE2 cross-reactive antibodies, mice or rabbits were immunized with recombinant SARS-CoV-2 S1-RBD produced by *E. coli*, mammalian cells (CHO cells) and insect cells (S2) emulsified with IFA or alum. Mice immunized with SARS-CoV-2 S1-RBD from *E. coli* or CHO cells produced significantly higher amounts of antibodies against ACE2 than those found in sera from control or SARS-CoV-2 nucleoprotein (NP)-immunized mice (**Figures 2A–C**). Nevertheless, the titers of antibodies against ACE2 (from >800 to >2550) were less than 1% of the titers of antibody against S1-RBD (from >64,000 to >256,000) in these S1-RBD immune sera (**Figure 2D**).

### mAbs 127 and 150 Specifically Recognize SARS-CoV-2 S1-RBD but not Other Coronavirus S1 Proteins

**Figure 3:**
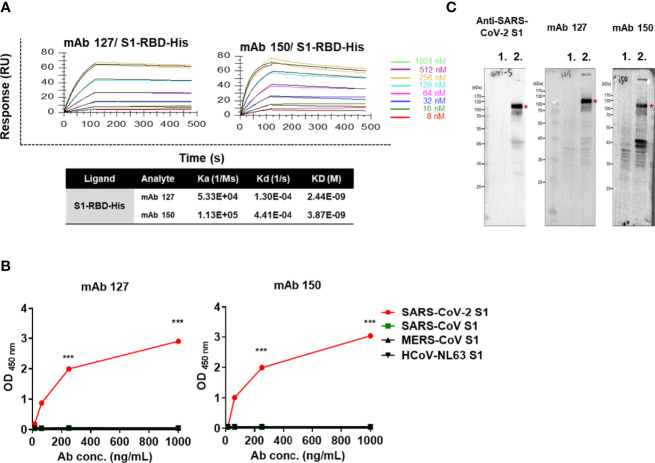
The mAbs 127 and 150 specifically bind to SARS-CoV-2 S1-RBD. **(A)** Affinity of mAbs 127 and 150 for S1-RBD. The S1-RBD-His-bound sensors were incubated with the different concentrations of mAbs 127 or 150 (indicated by different colors) for a set time interval to allow association. The sensors were then moved to protein-free solution and allowed to dissociate over a time interval. The binding kinetics were determined using Biacore T200 Evaluation Software version 3 with 1:1 binding model fitting. **(B)** The binding ability of mAbs 127 and 150 to SARS-CoV-2 S1, SARS-CoV S1, MERS-CoV S1 or HCoV-NL63 (1 μg/mL) was analyzed by indirect ELISA. **(C)** Cell lysates of mock-infected or SARS-CoV-2 NTU13-infected Vero-E6 cells (MOI=0.1, 24 h post-infection) were separated by SDS–PAGE under reducing condition and stained with 1 μg/mL commercial anti-SARS-CoV-2 S1 mAb or mAb 127 or 150 as indicated and visualized by Western blotting. Lane 1: lysates of mock-infected Vero-E6 cells; lane 2: lysates of NTU13-infected Vero-E6 cells. The asterisks indicate the SARS-CoV-2 S1 subunit of the S protein, which has a predicted MW of approximately 120 kDa. All statistical data are presented as the means ± S.Ds. from at least two independent experiments. ***P < 0.001.

To confirm that antibodies against S1-RBD can indeed cross-react with ACE2, about 200 hybridoma clones isolated from SARS-CoV-2 S1-RBD-immunized mice were generated. Among 21 candidates which could bind to SARS-CoV-2 S1-RBD, two mAbs, 127 (IgG2b) and 150 (IgG1) could bind to ACE2 as well. Both of these two mAbs did not show neutralizing activity against SARS-CoV-2 infection of Vero E6 cells *in vitro*. The binding affinity (KD) of mAbs 127 and 150 to S1-RBD was determined by Biacore™ SPR with S1-RBD-His-immobilized Sensor CM5 chips. The results showed that the binding affinities (KD) of mAbs 127 and 150 to S1-RBD were 2.44E-09 M and 3.87E-09 M, respectively (**Figure 3A**). Interestingly, although the amino acid identity among SARS-CoV-2 S1 protein and other coronavirus S1 protein are as high as 35~76%, both mAbs bound only to the SARS-CoV-2 S1 protein but not to the S1 proteins of SARS-CoV or other human CoVs (**Figure 3B**). In addition, the specific recognition of the authentic S1 subunit of the S protein of SARS-CoV-2 by mAbs 127 and 150 was confirmed by Western blotting analysis using cell lysates of SARS-CoV-2 (NTU-13)-infected Vero-E6 cells (**Figure 3C**). As shown in **Figure 3C**, a major band with a molecular weight (MW) of approximately 120 kDa, as predicted for the SARS-CoV-2 S1 subunit of the S protein, was recognized by the commercially available anti-SARS-CoV-2 S1 mAb and by mAbs 127 and 150. However, a higher number of nonspecific bands in cell lysates with or without virus infection were recognized by mAb 150 than by mAb 127.

### Characterization of the Binding Properties of mAbs 127 and 150 to ACE2

**Figure 4:**
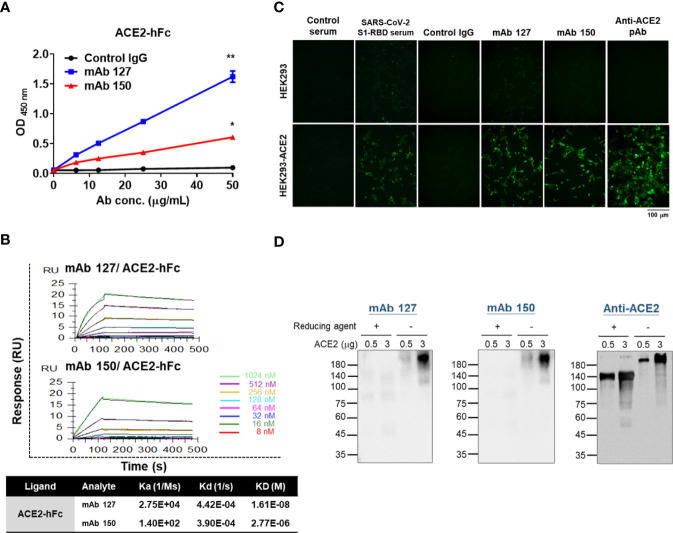
Characterization of the binding properties of mAbs 127 and 150 to ACE2. **(A)** The dose response of mAbs 127 and 150 and control IgG binding to ACE2-hFc was analyzed by indirect ELISA. **(B)** Binding affinity of mAbs 127 and 150 to ACE2. ACE2-hFc-bound sensors were incubated with the indicated concentrations of mAb 127 or 150 for a set time interval to allow association. The sensors were moved to protein-free solution and allowed to dissociate over a time interval. The binding kinetics were determined using Biacore T200 Evaluation Software version 3 with 1:1 binding model fitting. **(C)** HEK293 and HEK293-ACE2 cells were fixed and stained with control sera, SARS-CoV-2 S1-RBD-immunized sera (1:400), control IgG, mAb 127 or 150 or anti-ACE2 pAb (5 μg/mL) and then visualized with an immunofluorescent assay. **(D)** Western blotting analysis of mAb 127 or 150 or anti-ACE2 pAb binding to 0.5 or 3 μg of ACE2 in the presence or absence of a reducing agent as indicated. All statistical data are presented as the means ± S.Ds. from at least two independent experiments. *P < 0.05, **P < 0.01.

To characterize the binding ability of mAbs 127 and 150 to ACE2, an ELISA plate or SPR Sensor CM5 chips were coated with recombinant ACE2-hFc. The mAbs 127 and 150 could recognize recombinant ACE2-hFc in a dose-dependent manner (**Figure 4A**). In addition, the KDs of mAbs 127 and 150 to ACE2-hFc was 1.61E-08 M and 2.77E-06 M, respectively (**Figure 4B**). Furthermore, similar to the commercially available anti-ACE2 pAbs, mAb 127 and 150 and SARS-CoV-2 S1-RBD-immunized sera could recognize HEK293-ACE2 and showed a similar pattern to that obtained with the immunofluorescence assay (**Figure 4C**). However, the Western blotting analysis revealed that these mAbs could only recognize recombinant ACE2-hFc under nonreducing conditions migrated at ~210 kDa but not reducing conditions migrated at ~140 kDa (**Figure 4D**).

### Epitope Mapping of mAbs 127 and 150 on SARS-CoV-2 S1-RBD

**Figure 5:**
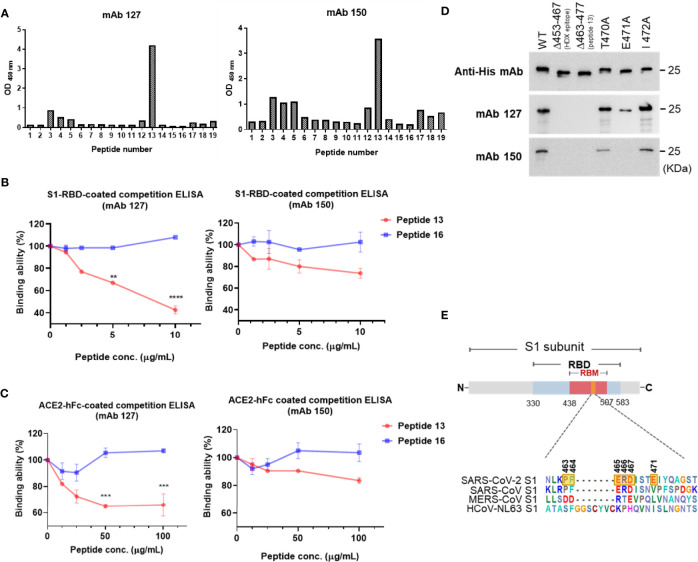
Epitope mapping of the ACE2-cross reactive sequence on SARS-CoV-2 S1-RBD. **(A)** The binding ability of mAbs 127 and 150 (0.5 μg/mL) to 19 different SARS-CoV-2 S1-RBD polypeptides (2 μg/mL) was analyzed by indirect ELISA. Competition ELISA showing the blockage of the binding of mAbs 127 and 150 to **(B)** SARS-CoV-2 S1-RBD or **(C)** ACE2-hFc by different doses of peptide 13 and control peptide 16. The binding ability (%) represents the percentage of preincubated peptides to antibodies compared with that obtained without peptide preincubation. **(D)** One hundred nanograms of wild-type (WT) and five different mutant His-tagged S1-RBD recombinant proteins, namely, HDX-MS sequence deletion (Δ453-467), peptide 13 deletion (Δ463-477), 470 mutant (T470A), 471 mutant (E471A), and 472 mutant (I472A), were separated by SDS–PAGE and then stained with 1 μg/mL anti-His mAb, mAbs 127 and 150. The binding ability was visualized by Western blotting. **(E)** The schematic sequence of the mAb epitope compared with the S1-RBD of four strains of CoV, namely, SARS-CoV-2 (YP_009724390.1), SARS-CoV (WH20 strain AAX16192.1), MERS-CoV (AFS88936.1), and HCoV-NL63 (APF29071.1), was aligned using BioEdit software. The epitope of mAb 127 is presented with six transparent yellow blocks (463, 464, 465, 466, 467, and 471) in the SARS-CoV-2 S1 sequence. All statistical data are presented as the means ± S.Ds. from at least two independent experiments. **P < 0.01, ***P < 0.001, ****P < 0.0001.

To map the epitope region of mAbs 127 and 150 on SARS-CoV-2 S1-RBD, nineteen overlapping S1-RBD polypeptides were synthesized (**Supplementary Figure 1**). The binding ability of mAbs 127 and 150 to each of these peptides was analyzed by ELISA. Our results showed that both mAbs 127 and 150 strongly binds to peptide 13 (PFERDISTEIYQAGS, a.a. 463-477) (**Figure 5A**). This result was further supported by an analysis of the antibody footprints obtained by HDX-MS analysis. After deuterium/hydrogen exchange, the S1-RBD protein was digested into several peptides by appropriate proteolytic digestion. Among these peptides, the peptide YRLFRKSNLKPFERD (a.a. 453-467) showed the highest H-D exchange in the presence of either mAb (**Supplementary Figure 2**), and this peptide shares an overlapping sequence with peptide 13. Based on these results, we speculated that part of the epitope recognized by mAbs 127 and 150 is located near a.a. 463-466 (PFERD). The epitopes of these mAbs were further delineated by peptide competitive ELISA. The results showed that the presence of peptide 13 but not the control peptide decreased the binding of these two mAbs to S1-RBD in a dose-dependent manner (**Figure 5B**). Similar competitive inhibition of the binding of mAb 127 and 150 to ACE2 was also observed in the presence of peptide 13 but not the control peptide (**Figure 5C**). To explore the proximity of the boundaries of the epitope on S1-RBD recognized by these mAbs, we individually changed three SARS-CoV-2 S1-RBD-specific amino acids downstream of the PFERD sequence, T470, E471, and I472, to alanine for further investigation. In addition, two deletion mutants were constructed: one was based on the HDX-MS (a.a. 453-467) results, and the other was based on the mapping results for peptide 13 (a.a. 463-477). The binding ability of these two mAbs to WT S1-RBD protein was compared with that of five different His-tagged mutant S1-RBD recombinant proteins, namely, the HDX-MS-determined epitope sequence deletion (δ453-467), the peptide 13 deletion (δ463-477), a 470 site-directed mutant (T470A), a 471 site-directed mutant (E471A), and a 472 site-directed mutant (I472A), by Western blotting analysis. The results showed that the mAbs 127 and 150 were unable to bind to either deletion mutant (**Figure 5D**). Additionally, compared with their binding abilities to WT S1-RBD, the binding ability of mAb 127 to the E471A mutant protein was reduced, and the binding of mAb 150 to the E471A mutant protein was abolished. In contrast, the binding ability of mAb 127 to the T470A and I472A mutant proteins was slightly decreased, even though the binding ability of mAb 150 to these two mutant proteins was slightly lower (**Figure 5D**). The binding abilities of mAbs 127 and 150 to these different SARS-CoV-2 S1-RBD mutant proteins were also confirmed by ELISA (**Supplementary Figure 3**). Because the a.a. 463-467 are conserved between SARS-CoV-2 and SARS-CoV, these results suggest that in addition to a.a. 463-467, a.a. 471 is also critical for the recognition of S1-RBD by both mAbs 127 and 150 (**Figure 5E**).

### Footprints of mAb 127 on S1-RBD and ACE2

**Figure 6:**
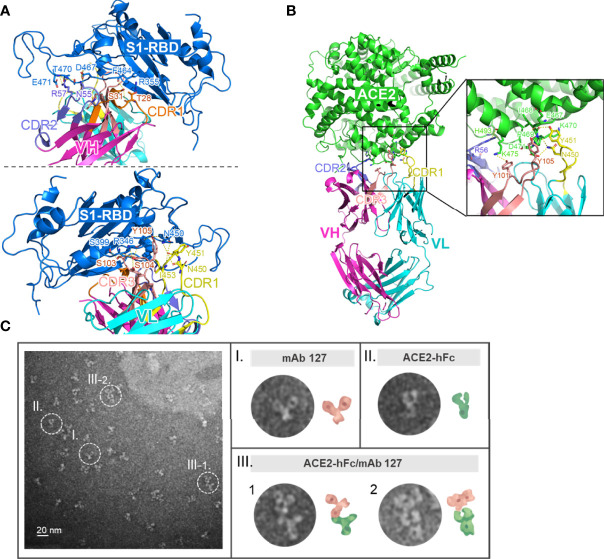
Footprints of mAb 127 binding to S1-RBD and ACE2. **(A)** A modeled structure of mAb 127 Fab/S1-RBD complex. mAb 127 Fab is generated by AlphaFold2 and the interaction is predicted by antigen-antibody docking. The interaction of the variable region of heavy chain (VH) (upper panel) and the light chain (VL) (lower panel) of mAb 127 with S1-RBD are visualized by Pymol. VH, magenta; VL, cyan; S1-RBD, blue. **(B)** A modeled structure of mAb 127 Fab/ACE complex. VH, magenta; VL, cyan; ACE2, green. **(C)** Negative-stain electron microscopy of samples containing mAb 127 (I) and ACE2-hFc (II). Representative two-dimensional class averages of ACE2-hFc bound by mAb 127 (III-1.2).

To investigate how these S1-RBD-specific mAbs cross react with ACE2, one of these two mAbs, mAb 127 was chosen to identify the protein–protein interaction between mAb and ACE2 by HDX-MS due to its higher binding affinity to ACE2 (**Supplementary Figure 4**). Among all ACE2 peptides, the peptide KGEIPKDQWMKKWWEM (a.a. 465-480), which is also located on the surface of ACE2, showed the highest H-D exchange in the presence of mAb 127. To confirm the footprints of mAb 127 on S1-RBD and ACE2, the amino acid sequence of the mAb 127 variable region was analyzed for further interaction prediction (**Supplementary Figure 5**). We used AlphaFold2 to build the 3D structure models of the Fab of mAb 127 to S1-RBD and ACE2. The binding regions between the mAb 127 Fab to S1-RBD or ACE2 were further analyzed and predicted by antibody-antigen docking (**Figures 6A, B**). As shown in **Figure 6A**, the interaction was predicted to occur between VH-CDR1, VH-CDR2, VL-CDR1, VL-CDR3, and a.a. 346, 355, 399, 450, 464, 467, 470, 471 of S1-RBD, which matched partial mapping result from S1-RBD HDX-MS (a.a. 453-467) and S1-RBD peptide 13 (a.a. 463-477). For docking on ACE2, a major chain interaction was predicted to occur on VL-CDR1, VH-CDR3, and a.a. 467-471 of ACE2. Side chain interactions were predicted on VH-CDR2, VH-CDR3, and a.a. 493 and 475 of ACE2 (**Figure 6B**). The docking interaction region was located within the same peptide region found by ACE2 HDX-MS (peptide KGEIPKDQWMKKWWEM). To visualize the interaction between ACE2 and mAb 127, freshly prepared mAb 127 and ACE2-hFc were coincubated prior to negative staining on grids. The negative-stained TEM analysis showed that mAb 127 bound to ACE2-hFc with one arm (**Figure 6C**). Collectively, these data suggest that S1-RBD-specific mAb 127 can bind to not only S1-RBD but also ACE2.

## Discussion

In this study, we confirmed that ACE2 cross-reactive antibodies were increased in the sera of some patients with COVID-19 and found a positive association between the amount of antibody binding to ACE2 and S1-RBD in sera from patients with COVID-19. Additionally, the antibody binding to ACE2 was significantly diminished in six out of 30 serum samples from patients with COVID-19 after preadsorption with S1-RBD. Interestingly, five of these six serum samples were collected from African American. However, the possible correlation between race and the production of ACE2 cross-reactive anti-S1-RBD antibodies should be further investigated. Nevertheless, these results suggest the presence of at least two different types of anti-ACE2 antibodies in patients with COVID-19. One type of these antibodies can cross-react with S1-RBD, which is probably induced by antigenic cross-reactivity between S1-RBD and ACE2 during SARS-CoV-2 infection. The other type of anti-ACE2 antibodies that cannot be adsorbed by S1-RBD preadsorption may be induced by other mechanisms which were discussed below. To further confirm that S1-RBD can indeed induce antibody cross-reactivity with ACE2, we immunized mice or rabbits with S1-RBD recombinant proteins generated from multiple sources, such as *E*. *coli*, mammalian cells and insect cells, and found that antibodies that cross-reacted with ACE2 could be detected in all these cases. In addition, two mAbs, 127 and 150, which could recognize both S1-RBD and ACE2, were isolated from SARS-CoV-2 S1-RBD-immunized mice. Even though the binding affinities of these two mAbs to ACE2 vs. S1-RBD were relatively low (10-1000 fold difference based on Biacore analysis), these results suggest a potential less-than-optimal structural mimicry between SARS-CoV-2 S1-RBD and ACE2.

Several different mechanisms have been proposed to explain the development of autoantibodies during SARS-CoV-2 infection. Dysregulation of the immune response during SARS-CoV-2 infection may lead to the breakdown of self-tolerance (30). Indeed, the activation of extrafollicular B cells, which share the B cell repertoire features previously described in autoimmune settings, has been found in critically ill patients with COVID-19 (31). Polyclonal B cell activation has also been detected in primary SARS-CoV-2 infection (32). In contrast, based on the theory of the idiotype and anti-idiotype network (33), a robust neutralizing anti-S1-RBD antibody response may induce an anti-idiotype antibody that can cross-react with the S1-RBD receptor ACE2 during SARS-CoV-2 infection (23). Alternatively, epitope spreading has also been proposed to explain the development of antibodies against ACE2 during SARS-CoV-2 infection due to the endocytosis of the complex of S protein and soluble ACE2 by macrophages (34, 35). In this study, we proposed molecular mimicry as another possible mechanism to explain the development of autoantibodies against ACE2 during SARS-CoV-2 infection. Molecular mimicry, which refers to sequence homology or structural similarity between molecules of the host and pathogens, is a common strategy used by viruses to counteract the immune response and evade immune recognition (36). Molecular mimicry has been proposed to explain the multiorgan damage observed in patients with COVID-19 (37–39). However, many of these reports are based on sequence homology between SARS-CoV-2 and host proteins and contain little clinical or experimental evidence supporting their findings. Here, we found that antibodies against S1-RBD in the sera of SARS-CoV-2-infected patients could also recognize ACE2. In addition, mAbs (127 and 150) which could recognize both S1-RBD and ACE2 were isolated from S1-RBD immunized mice. Although the binding affinities of these two mAbs to ACE2 vs. S1-RBD were low, these results suggest that the potential antigenic similarity between S1-RBD and ACE2 may induce ACE2 cross-reactive anti-S1-RBD antibodies during SARS-CoV-2 infection, which may represent one of the strategies used by SARS-CoV-2 to evade immune recognition.

In this study, a.a. 463-466 (PFERF) of S1-RBD recognized by mAbs 127 and 150 was identified using synthetic overlapping peptides and HDX-MS-based epitope mapping. The sequence of this epitope of S1-RBD on SARS-CoV-2 is distinct from other three CoVs, which shares less than 20% sequence identity. In addition, the SARS-CoV-2-specific a.a. 471 was identified by alanine substitution and sequence comparison of the S1-RBD between SARS-CoV-2 and SARS-CoV. It indicated that the a.a. 463-467 and 471 of the S1-RBD are critical and may imply the possible correlation between autoimmunity and unique pathology of SARS-CoV-2. The recognition of the authentic S protein of SARS-CoV-2 by mAbs 127 and 150 was confirmed by Western blotting analysis using cell lysates from SARS-CoV-2 (NTU-13)-infected Vero-E6 cells (**Figure 3C**). In addition to the S1 protein, some nonspecific bands were also recognized by mAb 150, which may be due to the lower affinity of mAb 150 to S1-RBD as compared with that of mAb 127 (3.87E-09 M vs. 2.44E-09 M, respectively). To our surprise, we found VL and VH genes of mAb 127 and 150 are the same as determined by 5’ RACE PCR. Since the isotypes of mAb 127 (IgG2b) and mAb 150 (IgG1) are different as defined by isotyping antibodies and isotype switching may cause the change of mAb affinity (40, 41), whether the decrease of the affinity of mAb 150 to S1-RBD is due to the isotype difference or other reasons remain unclear.

Different approaches were performed to understand the interaction between these mAbs and ACE2. Given that hFc fusion results in ACE2 dimerization (42), we found that mAbs 127 and 150 could only recognize native structure of ACE2-hFc in conditions without a reducing agent but not under reducing conditions as shown by Western blotting analysis. These results suggest the recognition of mAb 127 and 150 to ACE2 as compared to S1-RBD is more conformation-dependent. Negative-stained TEM images confirmed mAb 127 could bind to ACE2-hFc molecule with one of its arms. In addition, based on the HDX-MS analysis of the ACE2 heatmap recognized by mAb 127 and the docking model of mAb 127 on ACE2, we predicted the epitope recognized by the variable region of the light chain and heavy chain of mAb 127 on ACE2 was around a.a. 467-471. Interestingly, when we superimposed the epitopes of S1-RBD and ACE2 recognized by mAb 127 as predicted by AlphaFold2 on a S1-RBD/ACE2 complex 3D model (**Supplementary Figure 6**), we found that the regions of S1-RBD and ACE2 recognized by mAb 127 were different from the regions involved in the direct contact between S1-RBD and ACE2. This may explain why these S1-RBD specific mAbs cannot neutralize SARS-CoV-2 infection. However, more experiments such as crystal structure analysis of the mAb 127 Fab in complex with S1-RBD or ACE2 is required to fully define the precise interaction of mAb 127 and S1-RBD or ACE2.

Since the emergence of SARS-CoV-2 in late 2019, many mutations have emerged in different variants of concern (VOCs) to escape neutralization by antibodies (43, 44). Intriguingly, we found that the epitope recognized by mAbs 127 and 150 on S1-RBD is unique for SARS-CoV-2 among other CoVs, which shares only 0~50% similarity but highly conserved in all SARS-CoV-2 VOCs found thus far (43). Whether this finding indicates some evolutionary advantage for SARS-CoV-2 to preserve this structure remains unclear. Nevertheless, it indicates that antibody response induced by SARS-CoV-2 S1-RBD may play a dual role in protection and immunopathogenesis. Indeed, antibodies against SARS-CoV-2 S1-RBD which may drive significant complement activation and cellular inflammation that could have negative consequences during COVID-19 have been reported (45). In addition, higher anti-S antibody titers have been associated with worse clinical outcomes during SARS-CoV-2 infection (46). However, the pathogenic roles of ACE2-cross-reactive anti-RBD antibodies in COVID-19 infection remain to be explored.

Autoantibodies to ACE2 that can inhibit ACE2 activity have been found in patients with COVID-19 as well as patients with connective tissue diseases associated with vasculopathies (23, 47). In contrast, autoantibodies against ACE2 have been found to be correlated with elevated proinflammatory responses and increased COVID-19 severity (22). Moreover, IgM antibodies against ACE2 in sera from patients with COVID-19 may bind to ACE2-expressing tissues and activate complement to cause tissue damage (24). Therefore, in addition to the quantity of antibodies, the quality (such as the specificity, affinity, and isotype) of the anti-S antibodies may determine whether the antibodies are protective or pathogenic in patients with COVID-19 (30).

In summary, this study revealed potential antigenic cross-reactivity between SARS-CoV-2 S1-RBD and its receptor, ACE2, which could induce ACE2 cross-reactive antibodies during SARS-CoV-2 infection and in S1-RBD-immunized mice. Currently, several different types of SARS-CoV-2 vaccines have been developed, and some of them have been massively immunized in humans (48). For example, AstraZeneca (AZ) vaccine which is a SARS-CoV-2 vaccine based on a replication incompetent chimpanzee adenovirus expresses a native-like SARS-CoV-2 S glycoprotein (49). It is possible different structure and conformation of recombinant S1-RBD and native-like spike glycoprotein may contribute to the recognition of different epitopes of S1-RBD by B cells during S1-RBD immunization as reported here and AZ vaccination in humans (12–14). In addition, antibody response to SARS-CoV-2 vaccination can also be influenced by genetic background and immune status of different individuals. Therefore, whether ACE2 cross-reactive S1-RBD antibodies are induced after SARS-CoV-2 vaccination remains to be investigated (48). Nonetheless, the ACE2 cross-reactive S1-RBD mAbs found in this study provide valuable reagents to address the contribution of these ACE2 cross-reactive S1-RBD antibodies in the immunopathogenesis of COVID-19 during SARS-CoV-2 infection in the future.

## Data Availability Statement

The mAbs sequences presented in the study are deposited in figshare. (https://doi.org/10.6084/m9.figshare.19550272.v1).

## Ethics Statement

The studies involving human participants were reviewed and approved by IRB approved protocol (SDP-001-FDA Licensed Plasmapheresis Center; SDP-002- U.S. Physician Network; SDP-003 Reference Laboratory Network). Samples were purchased from Access Biologicals LLC. Written informed consent for participation was not required for this study in accordance with the national legislation and the institutional requirements. The animal study was reviewed and approved by the Institutional Animal Care and Use Committee (IACUC) under the number IACUC-10908006.

## Author Contributions

Y-CC, Y-WC, Y-CL, C-HC and T-MY conceived and designed the experiments. Y-CL, T-MY and Y-CC wrote and edited the paper. Y-CL, Y-YC, Y-CC, C-HC, Y-WC, Y-RW, L-YC, C-KT, C-KL, C-DC and W-JT performed the experiments and analyzed the data. SW, Y-SL, C-PC, W-JC, S-YC, WH and J-RW provided comments and suggestions during the preparation of the manuscript. All authors contributed to the article and approved the submitted version.

## Funding

This study was supported by the board of directors of Leadgene Biomedical, Inc., the Ministry of Science and Technology of Taiwan (MOST 109-2327-B-006-005, MOST 109-2327-B-006-007, MOST 109-2320-B-006-050, MOST 110-2320-B-006-033) and the National Health Research Institute (MR-110-CO-07).

## Conflict of Interest

Y-CL was employed by Leadgene Biomedical, Inc. Y-WC, Y-YC, L-YC, Y-RW, and Y-CC are employed by Leadgene Biomedical, Inc. C-DC is employed by OmicsLab Co., Ltd. C-KT and C-KL are employed by SIDSCO Biomedical Co., Ltd.

The remaining authors declare that the research was conducted in the absence of any commercial or financial relationships that could be construed as a potential conflict of interest.

## Publisher’s Note

All claims expressed in this article are solely those of the authors and do not necessarily represent those of their affiliated organizations, or those of the publisher, the editors and the reviewers. Any product that may be evaluated in this article, or claim that may be made by its manufacturer, is not guaranteed or endorsed by the publisher.
