# Supplementary material for: Antigenic Cross-Reactivity Between SARS-CoV-2 S1-RBD and Its Receptor ACE2
Source: Front Immunol. 2022 May 4;13:868724. doi: 10.3389/fimmu.2022.868724 (PMC9114768; doi:10.3389/fimmu.2022.868724)
Supplement: Supplementary file 1 [file DataSheet_1.docx]

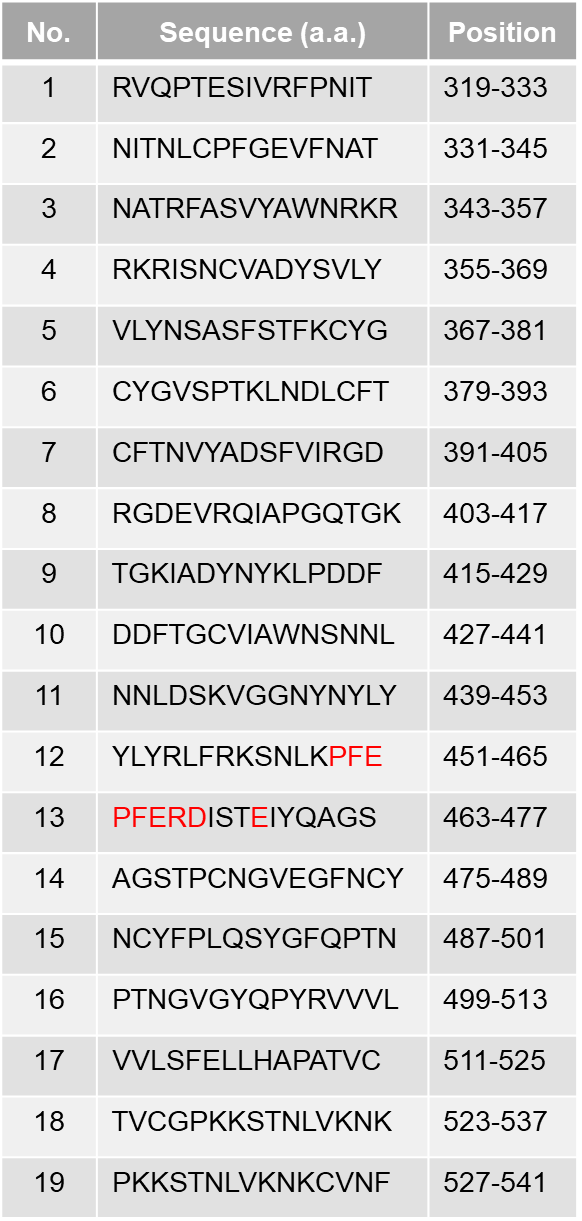


**Supplementary Figure 1. List of partially overlapping SARS-CoV-2 S1 RBD polypeptide amino acid sequences.** Nineteen overlapping polypeptides spanning the SARS-CoV-2 S1-RBD were synthesized to analyze the binding epitopes of mAbs 127 and 150. The projected critical epitopes recognized by mAbs 127 and 150 are highlighted in red.


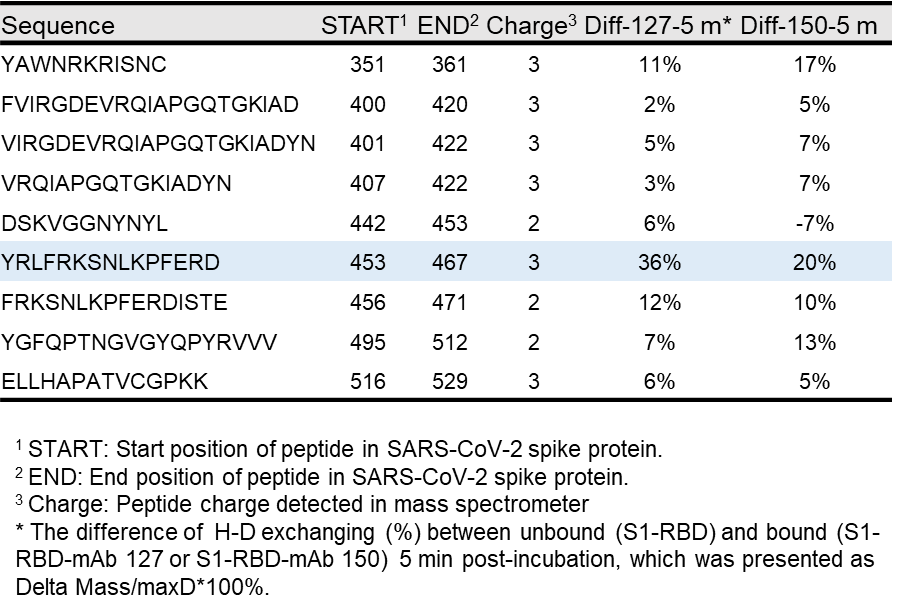


**Supplementary Figure 2. List of epitope mapping peptide sequences of S1-RBD recognized by mAbs 127 and 150 as determined by hydrogen-deuterium exchange mass spectrometry (HDX-MS).** The epitopes of S1-RBD recognized by mAbs 127 and 150 were determined by the relative deuterium uptake of overlapping peptides of S1-RBD over 5 min. The highest difference in H-D exchange (%) in either mAb 127-bound S1-RBD or mAb 150-bound S1-RBD is marked with light blue.


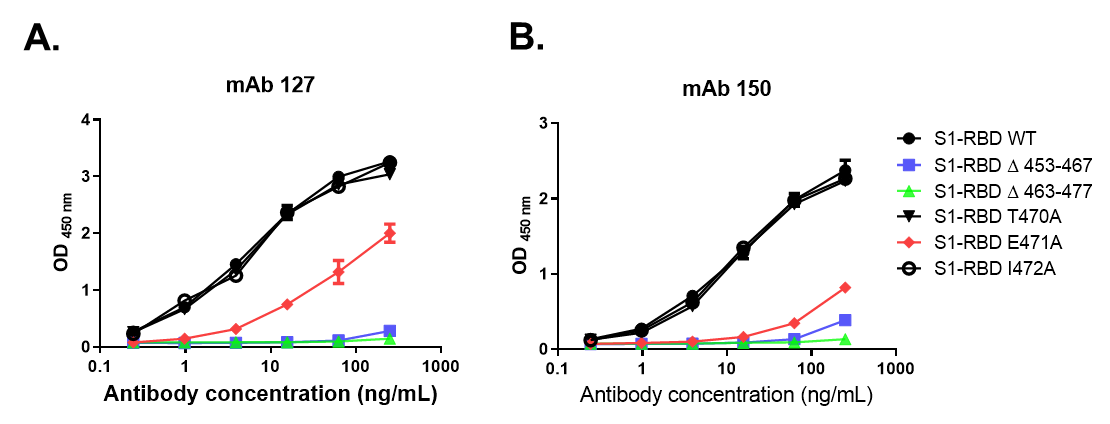


**Supplementary Figure 3. Binding ability of mAbs 127 and 150 to different mutant SARS-CoV-2 S1-RBD proteins.** The binding of different concentrations of mAbs 127 **(A)** and 150 **(B)** to wild-type (WT) and five different mutant His-tagged recombinant S1-RBD proteins (1 μg/mL) was analyzed by indirect ELISA.


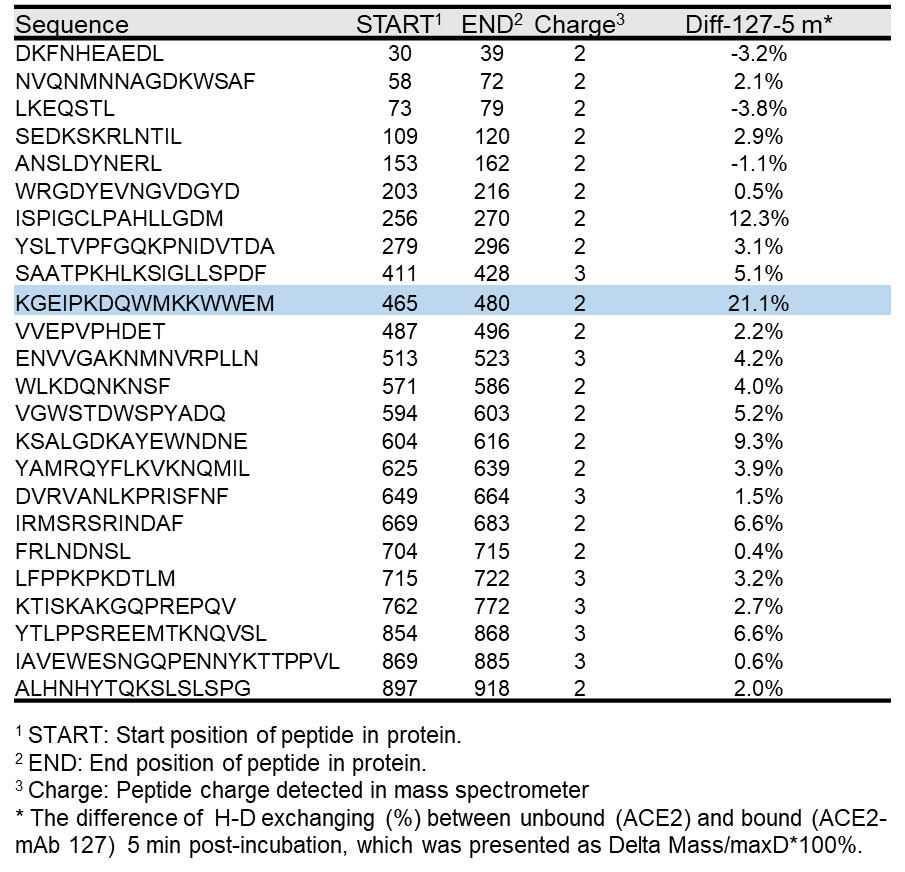


**Supplementary Figure 4. HDX-based epitope mapping of ACE2 recognized by mAb 127.** The epitopes of ACE2 recognized by mAb 127 were determined by the relative deuterium uptake of overlapping peptides of ACE2 over 5 min. The highest difference in H-D exchange (%) in mAb 127-bound ACE2 is marked with light blue.


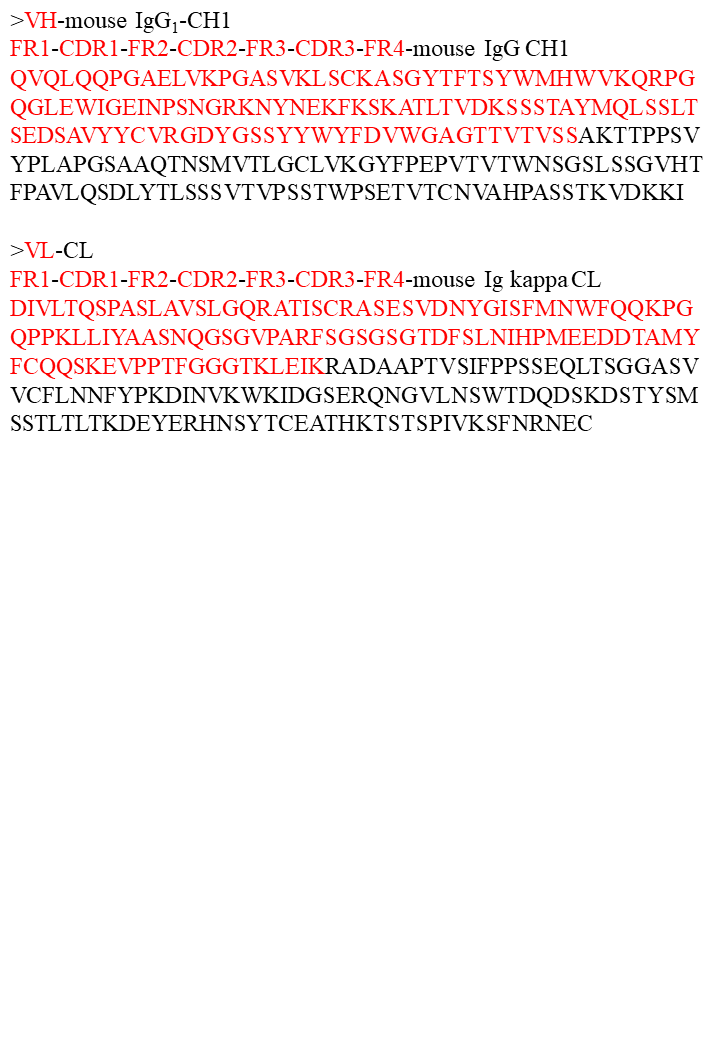


**Supplementary Figure 5. VH and VL amino acid sequence of mAb 127.** mRNA of mAb 127 hybridoma was extracted for V gene sequencing and analyzed. The variable region and constant region of both heavy chain and light chain were labeled in red and black, respectively.


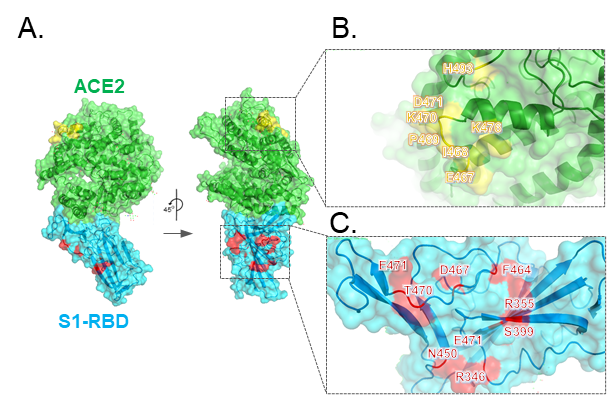
 **Supplementary Figure 6.** The predicted recognition sites of mAb 127 on a S1-RBD/ACE2 complex by Alphafold2. The crystal structure of the S1-RBD/ACE2 complex (PDB ID: 6M0J; green: ACE2; cyan: S1-RBD) was shown in cartoon and transparent surface presentation. The epitopes of ACE2 (yellow) and S1-RBD (red) recognized by mAb 127 shown are based on antibody-antigen docking result from Alphafold2. (B) and (C) are the ACE2 and S1-RBD structural models extracted from the S1-RBD/ACE2 complex model. The epitopes on ACE2 and S1-RBD were labeled with residues, respectively.
